# Investigation of the Propagation of Electrical Trees in a Polymer Matrix in the Corona Condition

**DOI:** 10.3390/polym10090984

**Published:** 2018-09-04

**Authors:** Chunyu Shang, Hui Sun, Yongqin Hao

**Affiliations:** 1 Department of Electrical and Information Engineering, Heilongjiang University of Science and Technology, Harbin 150022, China; zhuowen_1981@163.com; 2 National Key Laboratory of High Power Semiconductor Lasers, Changchun University of Science and Technology, Changchun 130022, China; celery1231@163.com

**Keywords:** polymer material, insulation application, corona resistibility, electrical tree

## Abstract

In a corona environment, the initiation and propagation of electrical trees in a polymer matrix originate from the field enhancement effect. Driven by the macroscopic alternating electric field, a weak alternating current (AC) was passed through the decomposition channel of an electrical tree, and a small amount of alternating electric quantity was present on the tip of the electrical tree, resulting in an enhanced local electric field around the tip of the electrical tree. The emissions of electrons accelerated in the enhanced local electric field resulted in the decomposition of the polymer material, stimulating the propagation of the electrical tree. When inorganic nano-particles with high corona resistibility were introduced into the polymer matrix, the nano-particles were aggregately deposited as the polymer material decomposed. The decomposition channel of the electrical tree was blocked and the current passing through the decomposition channel was shut off, eliminating the enhanced local electric field. As a result, the propagation of electrical trees was restrained and an improved corona resistibility was achieved for the polymer/nano-particles composite material.

## 1. Introduction

In electrical technology, especially in the high-voltage insulation field, polymer materials have been widely applied due to their excellent electrical, thermal, and mechanical properties [1,2,3,4,5,6]. There are many kinds of polymer materials, such as polyimide (PI), epoxy resin, polyethylene, cross-linked polyethylene, polystyrene plexiglass, and so on [7,8,9]. However, because the electrical erosion process takes a long time, electrical trees are propagated in a polymer matrix in the corona environment. The propagation of electrical trees in a polymer matrix is the typical pre-breakdown phenomenon leading to insulation failure, significantly shortening the life expectancies of polymer materials in insulation applications [10,11]. There has been extensive theoretical research conducted on the propagation mechanisms of electrical trees. Based on the inference that the molecular chains in polymer materials are destructed by high-energy particles or rays, Tanaka presented the theory of charge injection and extraction [1]. Kao K. C. and Tu D.M. proposed a trapping model of thermal electrons in polymer materials [12]. S. S. Bamji presented the theory of photodegradation [13]. Damamme et al. proposed that the energy release of trapped charges results in structural damage in polymer materials [14]. In addition, experimental investigations have been devoted to improving the corona resistibility of polymer materials, and some progress has been made, although the theoretical basis has been confusing and obscure for a long time. In this research paper, based on our comprehensive investigations, we present in theory that the initiation and propagation of electrical trees in a polymer matrix originate from the field enhancement effect. Based on the field enhancement mechanisms, we objectively investigated the microscopic corona processes on the surface and in the polymer matrix and precisely clarified the deflection and splitting dynamics of electrical trees in the polymer matrix. Because of the field enhancement mechanisms, we deliberately introduced inorganic nano-particles with high corona resistibility into the polymer matrix to restrain the initiation and propagation of electrical trees. By introducing inorganic nano-particles into the polymer matrix, we were able to verify that an improved corona resistibility had been achieved in the corona experiment. At the same time, we particularly present the mechanisms allowing for an improved corona resistibility for the polymer/nano-particles composite material, supporting the theory presented in this paper.

## 2. Experimental Section

Polyimide (PI) film and PI/TiO_2_ composite films (TiO_2_ content = 0 wt %, 1 wt %, 2 wt %, 3 wt %, 4 wt %, 5 wt %, 6 wt %, 7 wt %, 10 wt %, and 16 wt %) were synthesized via in situ polymerization. In order to prevent moisture absorption of the experimental materials, 4,4′-oxydianiline (ODA, supplied by Sinopharm Chemical Reagent Co., Ltd., Shanghai, China), pyromellitic dianhydride (PMDA, supplied by Sinopharm Chemical Reagent Co., Ltd.), and TiO_2_ nano-particles were heated at 100 °C for 8 h before the experiment. In an ultrasonic bath, *N,N*-dimethylacetamide (DMAC, supplied by Tianjin Fuyu Fine Chemical Co., Ltd., Tianjin, China) and TiO_2_ nano-particles with sizes of 30–100 nm (supplied by Beijing NaChen Technology Co., Ltd., Beijing, China) were added to a three-opening flask, and a stable suspension was formed after sufficient stirring and ultrasonic oscillation. A certain amount of ODA was added to the suspension. The three-opening flask was taken out of the ultrasonic bath and mechanically stirred for 20 min, waiting for the reactants to drop to room temperature. A certain amount of PMDA was added to the three-opening flask by stirring; the viscosity of the solution gradually increased, and the reaction was completed after 40 min. The three-opening flask was sealed and placed in a cool environment for 8 h, then vacuumed to remove the air bubbles. The mixture was cast on a glass substrate and scraped to ensure a uniform thickness. After being successively heated at 80 °C for 6 h, cured at 120 °C, 200 °C, and 300 °C for 2 h, PI film and PI/TiO_2_ composite films with a thickness of about 50 μm were obtained.

The XRD characterization of PI, TiO_2_, and PI/TiO_2_ composite material was identified by a Rigaku-D/Max 2500 X-ray diffractometer (Harbin, China) using Cu Ka radiation. The small angle X-ray scattering (SAXS) experiments were performed at the Small Angle Scattering Experimental Station in the Institute of High Energy Physics. The electron energy of the storage ring was 2.5 GeV and the average beam intensity was 120 mA. The size of the light spot on the sample was 1 mm × 3 mm, the X-ray energy range was 3–12 keV, and the wavelength range was 0.413–0.103 nm. The scattered signal was detected by the imaging plate detector (Mar3450, Harbin, China). The experimental data was normalized to obtain the scattering intensity of the sample. In the corona experiments, the corona aging system was mainly composed of the high-voltage AC power supply, the current-limiting resistor, the protection circuit, the electrodes and the electrode holders, etc. The high-voltage AC power supply consisted of a 0–220 V voltage regulator and a 220/4000 V high-frequency transformer. The main function of the current-limiting resistor was to limit the short-circuit current in the breakdown of the samples. The upper electrode was a columnar electrode with a diameter of 6.0 mm, a height of 150 mm, a chamfer radius of 1 mm, and a weight of 30 g. The lower electrode plate had a diameter of 30 mm and a chamfer radius of 2 mm. The electrode material was made of 310 stainless steel with a finish of 0.4 μm. In the experiments, the voltage and frequency applied were set at 1500 V and 3 kHz, respectively. For each of the PI/TiO_2_ composite films with different TiO_2_ contents, six samples with the same TiO_2_ content were taken in the time-to-breakdown measurements, and the average value of the six results was determined to be the final time-to-breakdown data. The morphologies of the PI/TiO_2_ composite films were characterized using a Quanta 200 environmental scanning electron microscope (ESEM, Harbin, China) operated at 20 kV with secondary and backscattering electrons in high-vacuum mode.

## 3. Results and Discussion

### 3.1. Microscopic Processes in the Initiation of an Electrical Tree in a Polymer Matrix

**Figure 1:**
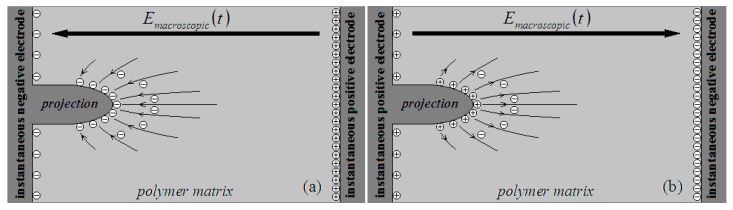
Schematic distributions of local electric field, free charges, and space charges around the top of the projection when $E_{macroscopic}\left(t\right)$ is (**a**) in the negative half-period and (**b**) in the positive half-period. The instantaneous polarities of the electrodes are labeled in the schematic diagram.

On a microscopic scale, the corona environment in which a piece of polymer material plays the role of insulation is complicated. In general, the electrode–polymer interface is not ideal: there may be a protrusion erected on a certain spot on the electrode surface (similarly, a conductive impurity particle on the electrode–polymer interface or a defect of a higher conductivity in the polymer surface), embedded in the polymer matrix [1]. In such a spot, and stimulated by the macroscopic alternating electric field $E_{macroscopic}\left(t\right)$ in the corona environment, a field enhancement effect is present around the projection top [14]. Moving toward the top, the enhanced local electric field $E_{local}\left(t\right)$ is inclined to be vertical to the projection surface. Moving away from the top, the field enhancement effect is gradually weakened, and $E_{local}\left(t\right)$ is gradually oriented to $E_{macroscopic}\left(t\right)$, as indicated in Figure 1.

In the negative half-period of $E_{macroscopic}\left(t\right)$ in the corona process, negative charges (electrons) are distributed on the top of the projection, trapped in the interface states. The enhanced local electric field $E_{local}\left(t\right)$ is oriented toward the top, as indicated in Figure 1a. For a trapped electron, a triangular potential barrier is present on the projection–polymer interface. According to quantum mechanics, the electron may penetrate the potential barrier, emitting into the polymer matrix due to the quantum tunneling effect [15,16,17,18]. On the other hand, for a space charge (electron) trapped in a localized state in a polymer matrix around the top of the projection, a quantum tunneling effect may also happen due to the enhanced local electric field in the localized state.

In the negative half-period of $E_{macroscopic}\left(t\right)$, when an electron is emitted from the top of the projection (or from the polymer matrix around the top), the electron should be accelerated by $E_{local}\left(t\right)$, being constantly scattered in the polymer matrix around the top. After losing the kinetic energy in the scattering process, the electron (or its relaying electron) should be finally trapped in a localized state, becoming a space charge in the polymer matrix, as indicated in Figure 1a.

In reality, the distance of an electron emitting from the top of the projection into the polymer matrix is rather limited (a short-range emission). Such a distance is negligible in contrast to the distance between the electrodes [18]. Consequently, the variation in potential distribution caused by the short-range emissions of electrons may be neglected. This means that no electrons would be supplied from the electrode to the top when some electrons leave the top, emitting into the polymer matrix. Therefore, the following relation may be given:

(1) $$Q_{free}\left(t\right)={Q^{\prime }}_{free}\left(t\right)+Q_{space}\left(t\right),$$

where $Q_{free}\left(t\right)$ is the electric quantity on the top of the projection when no electrons have been emitted into the polymer matrix and ${Q^{\prime }}_{free}\left(t\right)$ is the electric quantity left on the top when electrons with electric quantity $Q_{space}\left(t\right)$ have been emitted into the polymer matrix, becoming space charges. The loss of electric quantity on the top of the projection (from $Q_{free}\left(t\right)$ to ${Q^{\prime }}_{free}\left(t\right)$) and the accumulation of space charges in the polymer matrix bring about a shrinkage in the local electric field around the top. For instance, for a certain decreasing magnitude $\Delta \sigma_{free}\left(t\right)$ in surface density of electric quantity on a certain spot on the top of the projection, the corresponding decreasing magnitude in the local electric field adjoining the top may be given to be [15]:

(2) $$\Delta {E^{\prime }}_{local}\left(t\right)=\frac{\Delta \sigma_{free}\left(t\right)}{\varepsilon_{0}\varepsilon_{r}}.$$

In the positive half-period of $E_{macroscopic}\left(t\right)$ in the corona process, positive charges are distributed on the top of the projection and the enhanced local electric field $E_{local}\left(t\right)$ is oriented outward the top, as indicated in Figure 1b. The space charges (electrons) trapped in the localized states in the polymer matrix around the top may not be stable. An electron may be emitted from a localized state due to the quantum tunneling effect, being accelerated by $E_{local}\left(t\right)$ and constantly scattered in the polymer matrix, approaching the top until it (or its relaying electron) recovers onto the top.

In the positive half-period of $E_{macroscopic}\left(t\right)$, the electric quantity on the top of the projection is positive ($Q_{free}\left(t\right)$ > 0). When space charges ($Q_{space}\left(t\right)$ < 0) are present in the polymer matrix around the top, the opposite electric quantity ($-Q_{space}\left(t\right)$ > 0) is additionally supplied onto the top due to the electrostatic induction effect. As a result, the electric quantity on the top of the projection should be adjusted to ${Q^{\prime }}_{free}\left(t\right)$, ${Q^{\prime }}_{free}\left(t\right)=Q_{free}\left(t\right)-Q_{space}\left(t\right)$, and the relation given in Formula (1) is still reliable. Because of the existence of space charges $Q_{space}\left(t\right)$ and the additional electric quantity $-Q_{space}\left(t\right)$ on the top of the projection, the local electric field $E_{local}\left(t\right)$ around the top is additionally strengthened. For an increasing magnitude $\Delta \sigma_{free}\left(t\right)$ in surface density of electric quantity on a certain spot on the top of the projection, the corresponding increasing magnitude in the local electric field adjoining the top should be $\Delta {E^{\prime }}_{local}\left(t\right)=\Delta \sigma_{free}\left(t\right)/\varepsilon_{0}\varepsilon_{r}$, and the relation given in Formula (2) is still reliable.

In the corona process, the macroscopic alternating electric field $E_{macroscopic}\left(t\right)$ may be rather strong, bringing about an enhanced local electric field $E_{local}\left(t\right)$ around the top of the projection. Because of the high strength of $E_{local}\left(t\right)$ in the scattering process of an electron after being emitted, the accumulated electronic kinetic energy $E_{k}$ in its free path may be high enough to break the chemical bond in the polymer matrix, forming low-molecular decomposition products [16]. The decomposition of the polymer material around the top of the projection means the initiation of an electrical tree on the surface of the polymer matrix.

### 3.2. Propagation of Electrical Trees in a Polymer Matrix in the Corona Process

In the subsequent propagation of an electrical tree in the polymer matrix, the decomposition channel of the electrical tree may be seen as a conductive extension of the protrusion on the electrode surface [18,19]. Similar to the initiation of an electrical tree, the subsequent propagation of an electrical tree originates identically from the field enhancement effect.

Based on the experiments, it may be determined that there is a transition region between the electrical tree and the polymer matrix on the tip of the electrical tree. Driven by the macroscopic alternating electric field $E_{macroscopic}\left(t\right)$, a weak alternating current $I_{channel}\left(t\right)$ passed through the decomposition channel of the electrical tree and a small amount of alternating electric quantity $Q_{space}\left(t\right)$ of space charges was present on the tip of the electrical tree. As a result, an enhanced local electric field $E_{local}\left(t\right)$ was present around the tip of the electrical tree. For the typical cosine electric field $E_{macroscopic}\left(t\right)$, in a stable case, the electric quantity $Q_{space}\left(t\right)$ on the tip of the electrical tree and the current $I_{channel}\left(t\right)$ passing through the decomposition channel of electrical tree may be given to be [15]:

(3a) $$Q_{space}\left(t\right)=Q_{\max}\cdot \cos\omega t,$$

(3b) $$I_{channel}\left(t\right)=\frac{dQ_{space}\left(t\right)}{dt}=-\omega \cdot Q_{\max}\cdot \sin\omega t,$$

where ω is the angular frequency of $E_{macroscopic}\left(t\right)$. $Q_{\max}$ should be rather small and $I_{channel}\left(t\right)$ should be rather weak when ω is not very high. The emissions of electrons accelerated in $E_{local}\left(t\right)$ result in the decomposition of the polymer material on the tip of the electrical tree, stimulating the propagation of the electrical tree.

The above propagation dynamics of electrical trees is based on the prerequisite that the electrical tree, as a decomposition channel, possess a certain conductivity to sustain its propagation in the polymer matrix, and this may be possible in the corona condition in the polymer matrix [18,19,20,21,22]. For instance, in cross-linked polyethylene (XLPE), disordered graphitic carbon has been detected to be condensed in the main channels of the branch-pine electrical tree, the channel resistance has been determined to be less than 10 Ω/µm, and the electrical tree may be treated as a quasi-conductor [23].

### 3.3. Patterns of Electrical Trees in a Polymer Matrix in the Corona Process

With the corona stimulation, if the polymer matrix were an ideal uniform continuum, the decomposition channel of an electrical tree should propagate straight forward along the direction of $E_{macroscopic}\left(t\right)$. Nevertheless, there are various factors causing the decomposition channel to follow a tortuous path, to be split into different branches, causing it to present a tree-like pattern.

Particularly, when the corona resistibility of an impurity particle (e.g., an insulating inorganic particle) is higher than that of the polymer matrix and the impurity particle is present in front of a propagating electrical tree, the propagation of the electrical tree may be slowed down and deflected, avoiding the impurity particle. On the other hand, the corona resistibility of a defect region should be weaker in the polymer matrix. An electrical tree propagating through the vicinity of a defect region may be deflected, tending to pass through the defect region due to the higher propagation rate in this direction.

**Figure 2:**
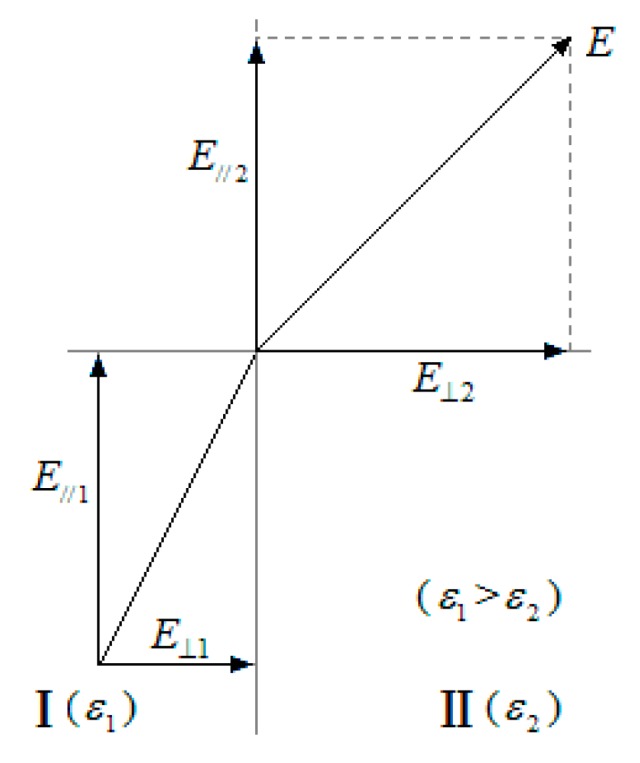
The differences in dielectric capacities in the microscopic structure lead to the deflection of the electric field in the polymer matrix.

Because of microscopic structural complexities, the dielectric capacity may not be constant in the polymer matrix, and the alternating electric field $E_{macroscopic}\left(t\right)$ should be weaker in a region with a higher dielectric constant ($\varepsilon_{r}$) and stronger in a region with a lower dielectric constant. As a result, the propagation rate of the electrical tree should be different, and the propagation of the electrical tree may be deflected. There is another factor causing the propagation of the electrical tree to be deflected when the dielectric constant is not consistent. As shown in Figure 2, when the instantaneous macroscopic electric field E is spreading across the interface of adjoined regions, I ($\varepsilon_{1}$) and II ($\varepsilon_{2}$), polarized charges would be present on the interface and the components of E on the interface should have the following relations [15]:

(4a) $$E_{//1}=E_{//2},$$

(4b) $$\varepsilon_{1}\cdot E_{⊥1}=\varepsilon_{2}\cdot E_{⊥2}.$$

The difference between $\varepsilon_{1}$ and $\varepsilon_{2}$ brings about the difference between $E_{⊥1}$ and $E_{⊥2}$, causing the electric field E to be deflected on the interface when E is not vertical to the interface [15]. As a result, the propagation of the electrical tree should be deflected due to the deflection of $E_{macroscopic}\left(t\right)$ in the polymer matrix.

In the propagation of an electrical tree, a decomposition channel may be split in different directions in the polymer matrix [24,25,26,27,28]. In a certain spot, an impurity particle or a defect may bring about the local anisotropic characteristics in the propagation rate of an electrical tree, causing the splitting of the electrical tree. In essence, the deflection and splitting dynamics of electrical trees in the corona process originates identically from the local anisotropic characteristics in the propagation rate of electrical trees, whereas the local anisotropic characteristics originate from the complexities in microscopic structures and compositions in the polymer matrix. Since the decomposition channel in the polymer matrix follows a tortuous path, being split in different spots, a tree-like pattern must be present in the corona process.

### 3.4. Improving the Corona Resistibility of a Polymer Matrix Based on the Propagation Mechanisms of Electrical Trees

In the corona environment, the propagation of electrical trees in the polymer matrix is the typical pre-breakdown phenomenon leading to the insulation failure, significantly shrinking the life expectancy of the polymer matrix in insulation applications [29].

**Figure 3:**
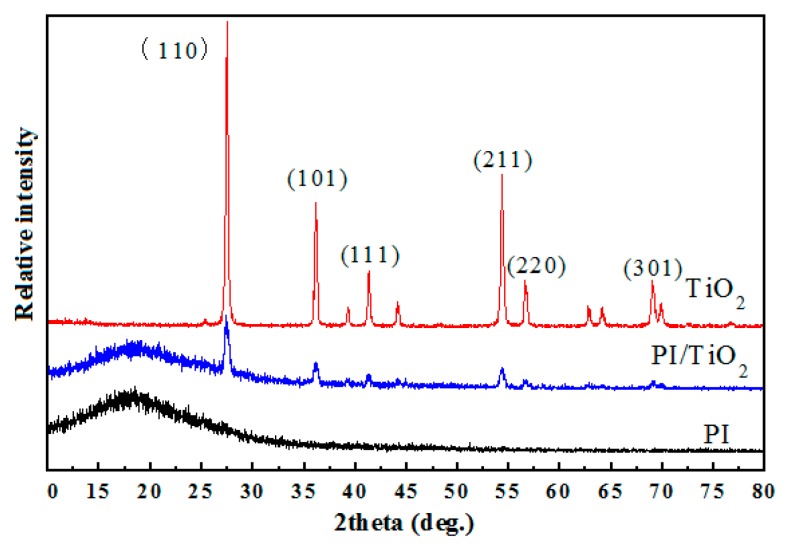
XRD diffraction data of polyimide (PI), TiO_2_, and PI/TiO_2_ composite material.

**Figure 4:**
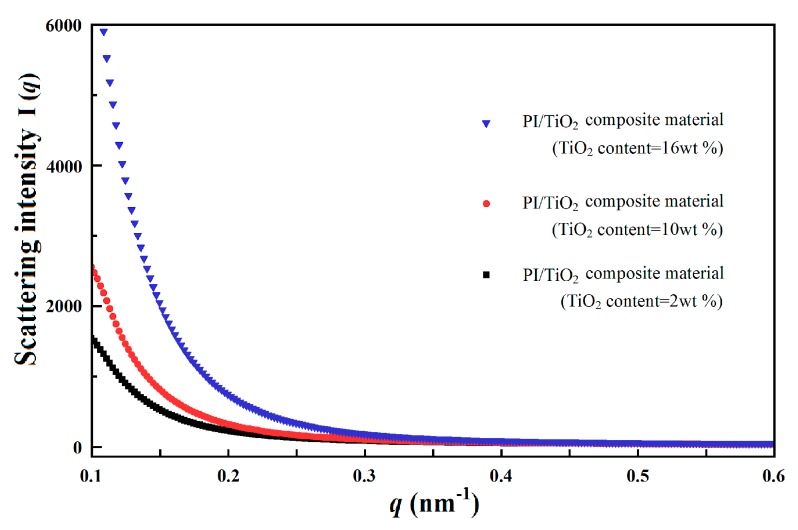
Small angle X-ray scattering (SAXS) experimental data for the PI/TiO_2_ composite material with different TiO_2_ contents.

**Figure 5:**
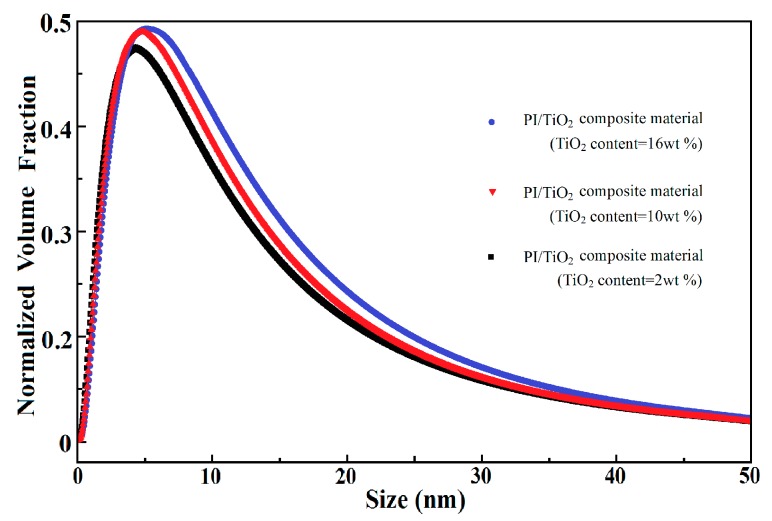
Size distributions of the TiO_2_ component in the PI/TiO_2_ composite material with different TiO_2_ contents.

**Figure 6:**
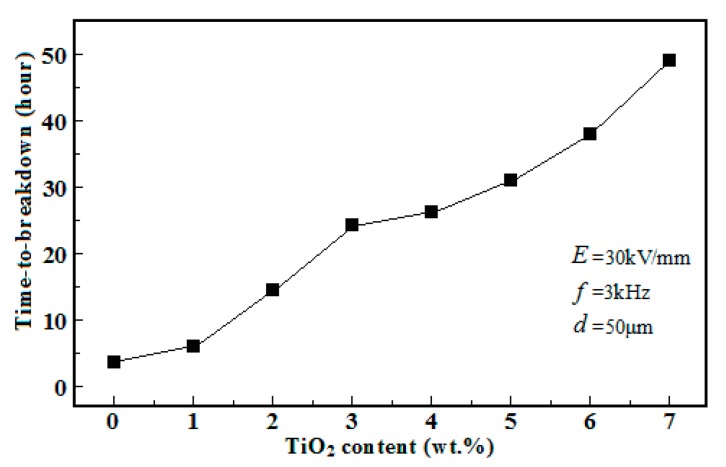
Time-to-breakdown data for PI/TiO_2_ composite films with different TiO_2_ contents.

Because of the propagation mechanisms of electrical trees, inorganic nano-particles with higher corona resistibility may be introduced into the polymer matrix to restrain the propagation of electrical trees. In the research undertaken, using polyimide (PI) as the typical engineering polymer material and titanium dioxide (TiO_2_) as the typical inorganic nano-particles, a PI/TiO_2_ composite material was formed and a series of experiments were carried out. The XRD diffraction data of PI, TiO_2_, and PI/TiO_2_ composite material are presented in Figure 3. Following the introduction of TiO_2_ nano-particles into the PI polymer matrix, the specific diffraction peaks of TiO_2_ were imposed on those of PI in the XRD diffraction data of the PI/TiO_2_ composite material. The SAXS experimental data for the PI/TiO_2_ composite material with different TiO_2_ contents are presented in Figure 4, where *q* represents the scattering wave vector and *I*(*q*) represents the scattering intensity. When *q* is smaller, the differences in scattering intensity *I*(*q*) are apparent for the PI/TiO_2_ composite material with different TiO_2_ contents, indicating that there are significant differences in electron-scattering characteristics between the TiO_2_ nano-particles and the polymer matrix. Based on the SAXS experimental data and the fitting of the continuous Gaussian distribution function, the size distributions of the TiO_2_ component were obtained for the PI/TiO_2_ composite material with different TiO_2_ contents, as presented in Figure 5. The size distributions of the TiO_2_ component did not significantly shift to the larger range with the increase of TiO_2_ content, indicating that the increase of TiO_2_ content did not result in the apparent agglomeration of TiO_2_ nano-particles in the polymer matrix. In the corona experiments, the time-to-breakdown data for the PI/TiO_2_ composite films with different TiO_2_ contents (0 wt %, 1 wt %, 2 wt %, 3 wt %, 4 wt %, 5 wt %, 6 wt %, and 7 wt %) were presented. The time-to-breakdown of the PI film was 3.9 h, whereas the time-to-breakdown of the PI/TiO_2_ composite film with a TiO_2_ content of 7 wt % was as high as 49 h, as indicated in Figure 6.

**Figure 7:**
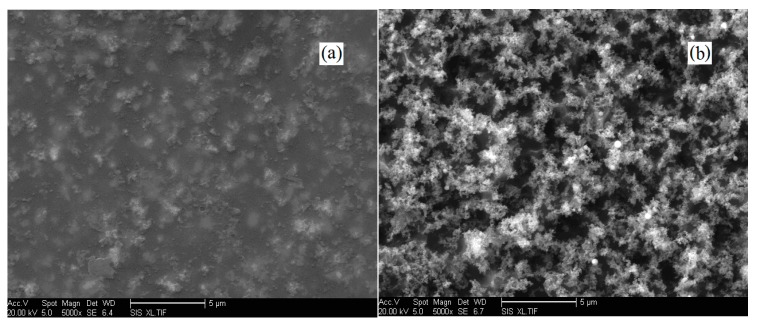
SEM morphologies of the PI/TiO_2_ composite films (**a**) before and (**b**) after the corona experiment.

On the basis of the propagation mechanisms of electrical trees presented in this paper, the corresponding interpretations may be clearly given. In the corona process, alternating current $I_{channel}\left(t\right)$ (Formula (3b)) would pass through the decomposition channel of the electrical tree, presenting the alternating electric quantity $Q_{space}\left(t\right)$ (Formula (3a)) on the tip of the electrical tree, stimulating the enhanced local electric field $E_{local}\left(t\right)$. For the polymer/nano-particles composite material, the decomposition of the polymer material would propagate tortuously forward into the composite matrix, avoiding the nano-particles due to the high corona resistibility that they possess. As a result, the polymer material around the nano-particles would be gradually decomposed and evaporated, leaving the nano-particles behind in the decomposition channel and on the surface of the composite matrix. In this case, the nano-particles would be aggregately deposited due to the high surface energy they possess. The decomposition channel of the electrical tree would be eventually blocked by the deposited nano-particles, shutting off the alternating current $I_{channel}\left(t\right)$ in the decomposition channel. Consequently, the propagation of the decomposition channel would be restrained in the corona process. In reality, in the corona process, following the decomposition of polymer material, the surface layer of the polymer/nano-particles composite matrix is gradually collapsed, leading to the deposition of nano-particles with disorganized morphologies. As a result, the initiation of electrical trees is restrained in the corona process. The morphologies of the PI/TiO_2_ composite films before and after the corona experiment are presented in Figure 7. Since the initiation and the subsequent propagation of electrical trees would be restrained in the corona process, the corona resistibility of the polymer/nano-particles composite material is inevitably improved.

As verified in the experiments and reported in the literature, inorganic nano-particles are more suitable than homogeneous inorganic micro-particles for introduction into the polymer matrix to restrain the initiation and propagation of electrical trees in the corona condition [30]. Based on the initiation and propagation mechanisms of electrical trees presented in this paper, the corresponding reasons can be precisely clarified. In the polymer/micro-particles composite material, following the decomposition of the polymer material propagating into the composite matrix in the corona process, inorganic micro-particles would be deposited in the decomposition channel and on the surface of the composite matrix. It should be noted that the interspaces between the deposited micro-particles should be of sub-micrometer magnitude, which are much larger than those in the deposited nano-particles. On the other hand, in typical polymer materials, the diameter size of an electrical tree ranges from a few tenths of a micrometer to tens of micrometers. Because of the relatively large interspaces between the deposited micro-particles, the decomposition channels of electrical trees may not be sufficiently blocked and the alternating current $I_{channel}\left(t\right)$ in the decomposition channel may not be sufficiently shut off in the corona process. As a result, the propagation of electrical trees may not be effectively restrained. Similarly, the initiation of electrical trees may also not be effectively restrained in the corona process. Consequently, the improvement in corona resistibility may not be sufficiently achieved by introducing inorganic micro-particles into the polymer matrix.

## 4. Conclusions

In this paper, the microscopic processes in the initiation and propagation of electrical trees in a polymer matrix were objectively investigated. Driven by the macroscopic alternating electric field in the corona process, a weak alternating current was passed through the decomposition channel of the electrical tree, and a small amount of alternating electric quantity was present on the tip of the electrical tree. As a result, an enhanced local electric field was present around the tip of the electrical tree. The emissions of electrons accelerated in the enhanced local electric field resulted in the decomposition of the polymer material, stimulating the propagation of the electrical tree. For the polymer/nano-particles composite material, the polymer material around the nano-particles was gradually decomposed in the corona process, leaving the nano-particles behind in the decomposition channel and on the surface of the composite matrix. The nano-particles were aggregately deposited, and the decomposition channel of the electrical tree was eventually blocked by the deposited nano-particles, shutting off the alternating current in the decomposition channel. As a result, the propagation of the electrical tree was restrained in the corona process. In the experiments, following the introduction of TiO_2_ nano-particles into the PI polymer matrix, an improved corona resistibility was achieved for the PI/TiO_2_ composite material.
